# Risk factors associated with *Salmonella* prevalence, its antibiotic resistance, and egg antibiotic residues in the layer farming environment

**DOI:** 10.14202/vetworld.2022.543-550

**Published:** 2022-03-09

**Authors:** Pairat Sornplang, Jareerat Aieamsaard, Chuleeporn Saksangawong, Naritsara Suayroop

**Affiliations:** 1 Division of Veterinary Public Health, Faculty of Veterinary Medicine, Khon Kaen University, Khon Kaen 40002, Thailand; 2 Division of Pharmacology and Toxicology, Faculty of Veterinary Medicine, Khon Kaen University, Khon Kaen 40002, Thailand; 3 Department of Animal Science, Faculty of Agriculture, Khon Kaen University, Khon Kaen 40002, Thailand

**Keywords:** antibiotic residue, antibiotic resistance, eggs, laying hens, risk factors, *Salmonella* prevalence

## Abstract

**Background and Aim:**

Human salmonellosis with non-typhoidal *Salmonella* remains a global public health concern related to the consumption of contaminated eggs and egg-based products. This study aimed to examine the prevalence of *Salmonella*, antimicrobial-resistant *Salmonella*, and egg antibiotic residues concerning risk factors associated with *Salmonella* contamination in eggs, the layer farming environment, and laying hens kept in battery-cage closed-housing systems.

**Materials and Methods:**

This study used a repeated cross-sectional design to collect 488 samples from eggs, laying hens, and the farm environment on one laying farm for *Salmonella* detection according to ISO 6579:2002/AMD 1:2007. *Salmonella*-positive samples were further tested for serotype and antimicrobial susceptibility using the disk diffusion test. The layer farm contact person was interviewed at the sampling time to evaluate the risk factors associated with *Salmonella* contamination using logistic regression analysis. For each month, 24 eggs (144 eggs in total) were also randomly sampled from the collection egg area at the farm for antibiotic residue detection using the European Four Plate Test.

**Results:**

The highest *Salmonella* prevalence rates were in the samples from the layer pen floors, followed by the egg sizing machine (ESM) and eggshells at 65.5%, 52.5%, and 15%, respectively. *Salmonella* *enterica* serovar Corvallis was the dominant serovar (48.38%), followed by Mbandaka (37.76%), Braenderup (14.29%), and Typhimurium (4.08%). Rodent presence at the farm and the frequency of changing the disinfectant foot dip were significant factors related to *Salmonella* contamination on the pen floors (odds ratio [OR]=22.5, 95% confidence interval [CI]=2.11-240.48, p=0.01; OR=24, 95% CI=2.78-206.96, p=0.004, respectively). Hand-washing before sorting and cleaning the ESM were the significant factors (OR=13, 95% CI=1.2-140.73, p=0.04). The most resistant *Salmonella* isolates were resistant to oxytetracycline. One isolate of *S*. *enterica* Typhimurium was resistant to cefotaxime, enrofloxacin, and oxytetracycline. The antibiotic residues in the egg yolks were streptomycin, enrofloxacin, and tetracycline at prevalence rates of 36.11%, 11.81%, and 7.64%, respectively. Streptomycin was the most abundant residue in the albumen and yolk, followed by tetracycline.

**Conclusion:**

*Salmonella* prevalence in layer farming with a closed-housing system is related to effective biosecurity and hygiene issues, such as rodent control, clean farm equipment, and good worker hygiene. In addition, eggs’ antibiotic residues may be related to treating antimicrobial-resistant *Salmonella* isolates and medicated feed with inappropriate antibiotic withdrawal time.

## Introduction

*Salmonella* remains a huge problem in poultry farming, and it is one of the major pathogens causing asymptomatic poultry salmonellosis, illness, or death. In addition, *Salmonella* also causes poultry product loss, such as weight loss in eggs and meat and laying reduction. Poultry meat and eggs are the main sources of *Salmonella* contamination, causing human salmonellosis worldwide. Human infection with non-typhoidal *Salmonella* remains a global public health concern related to poultry farm management and non-effective biosecurity programs. Laying hens and table eggs can bring about non-typhoidal *Salmonella* contamination in two ways: Vertical transmission from infected breeders and horizontal transmission from environmental sources, such as contaminated equipment, dust, feed, or drinking water [1,2].

*Salmonella* serotypes occurrence varies depending on hosts, environment, and areas. *Salmonella enteritidis* is commonly found in the egg industry in the United States, the United Kingdom, and China, while in Australia and New Zealand, the Typhimurium serovar has been the main pathogen found in eggs and egg products [3,4]. Recently, table eggs contaminated with serovar Hessarek, when stored at 25°C or higher, caused a *Salmonella* outbreak in humans in South Australia [5]. There is little information regarding egg safety and *Salmonella* serovar prevalence in the egg industry in Thailand.

Layer farming in conventional battery cages is the typical housing system used in commercial egg production but increases layer welfare problems. Free-range, organic, and conventional farming are alternatives to battery cages for laying hens [6]. A closed-laying farm system with an evaporative cooling system is one of the alternative ways to reduce the air temperature, especially during summer, leading to decreases in heat stress and improvement in egg production. This farming system enables convenient management in smart layer farming, such as automatic feeding and egg collection using the belt system. Little is known about the *Salmonella* prevalence in eggs and laying hens in battery cages adapted with an evaporative cooling housing system.

This study aimed to evaluate *Salmonella* prevalence and its possible contamination in layer farming at the modern Khon Kaen University (KKU) layer farm in Thailand.

## Materials and Methods

### Ethical approval and Informed consent

The Institutional Animal Care and Use Committee of KKU, based on the Ethics of Animal Experimentation of the National Research Council of Thailand, approved this study’s experimental animal procedure (IACUC-KKU 140/64). Before the interview (by the researchers), all participants read and understood the consent statement provided in the consent form in this study.

### Study period and location

We collected the laying hens, eggs, and environment samples related to layer farms at the modern KKU layer farms, Faculty of Agriculture, KKU, Thailand, from August 2020 to June 2021. The samples were analyzed for *Salmonella* detection at the Division of Veterinary Public Health, Faculty of Veterinary Medicine, KKU, Thailand.

### Study design and sample collection

We completed the sampling using a repeated cross-sectional design for 8 months, taking samples 3 times a month. The samples in this study included laying hens, eggs, eggshells, feed, drinking water, tap water, and surface samples related to the layer hen farm. The case study location was a smart KKU battery-cage layer farm modified with an evaporative cooling system, automatic feeding, and an egg collection belt system in Thailand. The farm consisted of 20,016 Hy-Line Brown laying hens (from CPF Public Co., Ltd., Thailand) of 17 weeks of age that lay from week 19 to week 91. They laid 92% on average. The laying hens were raised in standard conventional battery cages with five hens per cage (0.18 m^3^/hen). Each month, approximately ten samples from each laying hen, eggs inside the layer pen, and eggs at the egg sizing machine (ESM; Riva Selegg, Italy) were taken. In addition, the surface samples from different environmental sources included the layer pen floors, egg belts (EBs), and ESM. The total number of samples was 488. Four 25-cm^2^ representative positions of each of the following surfaces were swabbed for the surface samples: Pen floor, EB, and ESM leading to a total sample surface area of 100 cm^2^. Swabbing was performed using sterile gauze moistened with Buffered Peptone Water pH 7.2 (BPW; Oxoid, UK), forceps, and a 25 cm^2^ steel frame. Each surface sample was placed in a sterile sampling bag (3M Co., Ltd., Thailand) with 20 mL BPW. All 488 samples were kept cool at 4°C in a cool box with ice packs and transported to the bacterial laboratory for *Salmonella* examination within 12 h. *Salmonella* detection was conducted at the Division of Veterinary Public Health, Faculty of Veterinary Medicine, KKU, Thailand.

### Sample preparation, *Salmonella* isolation, and serotyping

In a pre-enrichment step of *Salmonella* detection, all samples were processed as follows: The cloacal swab samples from the layers were diluted in 10 mL BPW in ready-to-use swab tubes (3M Co., Ltd.); 25 g each of the feed, water, and egg samples were diluted in 225 mL BPW; and three egg samples were soaked in 30 mL BPW for analysis of one eggshell sample. All surface samples were diluted in sterile plastic sampling bags (3M Co., Ltd.), followed by the addition of 100 mL BPW. All BPW homogenate samples were incubated for 18-24 h at 37°C.

The detection of *Salmonella* spp. was further carried out under ISO 6579:2002/AMD 1:2007. Briefly, 100 mL-aliquots of the pre-enrichment sample were either inoculated with three pipette drops onto Modified Semisolid Rappaport Vassiliadis Medium (MSRV; Difco, USA) or into 10 mL of Muller Kauffmann Tetrathionate broth (TT; Oxoid) and then incubated at 42°C and 37°C, respectively, for 18-24 h. After enrichment, one loop of MSRV and TT cultures was streaked onto Xylose-Lysine-Deoxycholate (Oxoid) agar plate as a selective solid medium and incubated at 37°C for 18-24 h. This repeating step of the MSRV or TT cultures was streaked onto chromogenic agar of 3M Petrifilm™ *Salmonella* Express System (3M Co, Ltd.). Up to five typical *Salmonella* colonies per plate were selected to confirm *Salmonella* biochemically (such as using hydrogen sulfide, lysine, indole, lactose, and urease tests). Selected one or two confirmative *Salmonella* colonies were grown on nutrient agar (Oxoid) to send for *Salmonella* serotyping according to the White-Kauffmann-Le Minor scheme by slide agglutination with O and H antigen-specific sera (SAP laboratory Co., Ltd., Thailand).

### Questionnaire design

The person related to the layer farm (i.e., workers, manager, and visitors) was interviewed and observed through a structured questionnaire at the time of sampling time, 3 times/month for 8 months. The questionnaire was designed using a literature study about *Salmonella* contamination in poultry. The question was related to the workers, farm equipment, farm management, flies/insects, and rodents, and it was pre-tested and adjusted accordingly.

### Antimicrobial susceptibility test

All *Salmonella*-positive samples were tested for antimicrobial resistance using the Kirby–Bauer disk diffusion method [7]. The resistance of *Salmonella* serovars was interpreted using the Clinical and Laboratory Standards Institute guideline [8]. Four antibiotics that are commonly used for the treatment of human or animal infections were chosen to test their varied antibacterial activities: ampicillin (10 mg), cefotaxime (30 mg), enrofloxacin (5 mg), and oxytetracycline (30 mg). All antibiotic disks (diameter=6 mm) were obtained from Oxoid. *Escherichia coli* ATCC25922 was used as the comparable standard strain.

### Detection of antibiotic residue in egg samples

One hundred and forty-four egg samples with weight varying from 45 g to >70 g were sampled from the smart KKU layer farm from November 2020 to April 2021. All samples were separated into yolk and albumen for analysis of antibiotic residues using the European Four Plate Test, according to Kilinc and Cakli [9], and modified with the additional medium plate of *E. coli* seeding for the detection of fluoroquinolone residues. Five different inoculated media were tested for antibiotic detection. Bacterial suspensions of approximately 10^4^-10^5^ colony-forming unit (CFU)/mL of standard *Bacillus* *subtilis* were seeded onto three Mueller Hinton Agar (MHA; Becton Dickinson, USA) plates with three different pHs of 6, 7.2, and 8 (medium I, II, and III, respectively). The other two MHA plates were seeded with standard *Micrococcus luteus* and *E. coli* (approximately 10^4^-10^5^ CFU/mL) had a pH of 8 and 6 (and were medium IV and V, respectively). The MHA plate with a pH of 7.2 had added trimethoprim for increasing the sensitivity to sulfonamide residues. Antibiotic standards of the six different representative antibiotic families used in this study purchased from Oxoid were seeded onto the five different media: Penicillin G and tetracycline onto medium I; sulfamethoxazole onto medium II; streptomycin onto medium III; erythromycin onto medium IV; and enrofloxacin onto medium V. The separated egg (i.e., the albumen and yolk samples) was homogenized using vortexing. Ten microliters of each sample were dropped onto paper disks. After drying the disks at 40°C for 10 min, they were placed on the five previously mentioned medium plates and incubated overnight at 37°C. Interpretation of antibacterial potency of the samples used the guidance of the inhibition zone size of one or both microorganisms. The positive samples showed an inhibition zone (including the 6 mm of the disk diameter zone) ≥8 mm in diameter, while a zone <8 mm in diameter was considered negative; the inhibition zone of the standard antibiotic disk must be ≥24 mm in diameter.

### Limit of detection for different antibiotics

The stock solutions were prepared at 0.0320 mg/disk for penicillin, 0.0320 mg/disk for tetracycline, 0.1000 mg/disk for sulfamethoxazole, 1.0240 mg/disk for streptomycin, 0.0640 mg/disk for erythromycin, and 0.5120 mg/disk for enrofloxacin. These stock solutions were diluted using a two-fold dilution assay with five dilutions for each antibiotic. The lowest dilution showing the inhibition zone before the dilution of no inhibition zone indicated the antibiotics’ detection limit.

### Statistical analysis

The prevalence of *Salmonella* contamination in the different samples and the antibiotic residue in the egg samples was measured using descriptive statistics. Univariate logistic regression was performed for each categorical variable regarding the possible risk factors of *Salmonella* contamination. The significance level was set at p<0.05. Statistical Package for the Social Sciences (SPSS) statistical package (version 26.0, SPSS Inc., USA) was used to analyze the data.

## Results

### *Salmonella* prevalence and serotyping

Ninety-eight out of 488 samples were positive for *Salmonella*, and the prevalence among the pen floors, ESM, ESM eggshells, EB, tap water, EB eggshells, and laying hens were 62.50% (50/80), 52.50% (21/40), 15.00% (6/40), 13.75% (18/70), 12.50% (2/16), 7.50% (3/40), and 6.25% (5/80), respectively, as shown in Table-1. Table-2 shows the serotyping of *Salmonella* positive samples. The predominant serotype found in this study was *Salmonella enterica* serovar Corvallis, followed by Mbandaka, Braenderup, and Typhimurium at the prevalence rates of 43.88% (43/98), 37.76% (37/98), 14.29% (14/98), and 4.08% (4/98), respectively.

**Table-1:** *Salmonella* positive samples from the different samples from the smart Khon Kaen University layer farm.

Sample type	Monthly sampling (M^1^, Number of positive/Number of samples)								Total (%)

	M 1	M 2	M 3	M 4	M 5	M 6	M 7	M 8	
Pen floors	(1/3)	(1/3)	(3/3)	(2/3)	(5/8)	(10/20)	(10/20)	(18/20)	50/80 (62.50)
ESM^2^	(0/1)	(0/1)	(1/1)	(1/1)	(3/9)	(5/9)	(5/9)	(6/9)	21/40 (52.50)
ESM eggshells	(0/14)	(0/4)	(0/5)	(0/6)	(1/2)	(1/3)	(2/3)	(2/3)	6/40 (15.00)
EB^3^	(0/3)	(0/3)	(0/3)	(0/3)	(1/8)	(3/20)	(3/20)	(4/20)	11/80 (13.75)
Tap water	(0/2)	(0/2)	(0/2)	(0/2)	(0/2)	(0/2)	(1/2)	(1/2)	2/16 (12.50)
EB eggshells	(0/13)	(0/5)	(0/4)	(1/6)	(0/3)	(0/3)	(1/3)	(1/3)	3/40 (7.50)
Laying hens	(0/9)	(0/9)	(1/9)	(0/9)	(0/11)	(1/11)	(1/11)	(2/11)	5/80 (6.25)
Drinking water	(0/2)	(0/2)	(0/2)	(0/2)	(0/2)	(0/2)	(0/2)	(0/2)	0/16 (0.00)
Feed	(0/2)	(0/2)	(0/2)	(0/2)	(0/2)	(0/2)	(0/2)	(0/2)	0/16 (0.00)
Eggs	(0/27)	(0/9)	(0/9)	(0/12)	(0/5)	(0/6)	(0/6)	(0/6)	0/80 (0.00)
Total (%)	1/76 (1.32)	1/40 (2.50)	5/40 (12.50)	4/46 (8.70)	10/52 (19.23)	20/78 (25.64)	23/78 (29.48)	34/78 (43.59)	98/488 (20.08)

1 M1-M8, monthly sampling for 8 months during August 2020–June 2021

2 Egg sizing machine

3 Egg belt

**Table-2:** Serotyping of *Salmonella* positive samples.

*Salmonella* positive samples (n)	Serotypes (%)			

	Corvallis	Mbandaka	Braenderup	Typhimurium
Pen floors (50)	30 (60.00)	15 (30.00)	5 (16.67)	-
ESM^1^ (21)	6 (28.57)	10 (47.62)	5 (23.81)	-
ESM eggshells (6)	1 (16.67)	4 (66.67)	1 (16.67)	-
EB^2^ (11)	4 (36.36)	5 (45.45)	2 (18.18)	-
Tap water (2)	-	-	-	2 (100)
EB Eggshells (3)	2 (66.67)	1 (33.33)	-	-
Laying hens (5)	-	2 (40)	1 (20)	2 (40)
Total (98)	43 (43.88)	37 (37.76)	14 (14.29)	4 (4.08)

1 Egg sizing machine

2 Egg belt

### Antimicrobial susceptibility

The antimicrobial resistance prevalence of the *Salmonella* serovars to the four antibiotics tested is shown in Table-3.

**Table-3:** Antimicrobial resistance prevalence of the *Salmonella* positive samples.

*Salmonella* serovars (Number of positive)	Antimicrobial resistance agents^1^ (%)			

	AMP	CTX	ENR	OT
Corvallis (43)	0 (0)	0 (0)	0 (0)	20 (46.51)
Mbandaka (37)	0 (0)	0 (0)	0 (0)	10 (27.03)
Braenderup (14)	0 (0)	0 (0)	0 (0)	4 (28.57)
Typhimurium (4)	0 (0)	1 (25)	2 (50)	1 (25)
Total (98)	0 (0)	1 (1.02)	2 (2.04)	35 (35.71)

1 AMP=Ampicillin, CTX=Cefotaxime, ENR=Enrofloxacin, OT=Oxytetracycline

### Risk factor assessments

Tables-4-6 show the possible risk factors for *Salmonella* contamination on the laying farm on the pen floor, laying hen, and eggshell samples, respectively. There were two significant factors related to *Salmonella* contamination on the layer pen floors: Rodent presence at the farm (odds ratio [OR]=22.5, p=0.01) and the frequency in changing of the foot-dip (OR=24, p=0.004). For the ESM eggshells, hand-washed before sorting eggs and the ESM cleaning were significant factors in *Salmonella* contamination (OR=13, p=0.04; OR=13, p=0.04, respectively).

**Table-4:** The possible risk factors of *Salmonella* contamination on the layer pen floors from the smart Khon Kaen University layer farm.

Factor	*Salmonella* positive/tested	Prevalence (%)	Adjusted odds ratio	95% CI	p-value
Pen floor washed (at least 2 times/month)					
Yes	8/12	66.67	0.50	0.10-2.60	0.41
No	6/12	50.00			
Layer carcasses disposal (within 24 h)					
Yes	5/8	62.50	0.77	0.14-4.39	0.77
No	9/16	56.25			
Presence of rodents at the farm (at least 3 times/month)					
Yes	10/11	90.91	22.50	2.11-240.48	0.01
No	4/13	30.77			
Changing of foot-dip (at least 3 times/month)					
Yes	2/10	20.00	24.00	2.78-206.96	0.004
No	12/14	85.71			
Having boot-disinfection bath (at least for 5 months)					
Yes	8/15	53.33	1.75	0.31-9.75	0.52
No	6/9	66.67			
Layer waste management (every week)					
Yes	4/8	50.00	1.20	0.23-6.93	0.83
No	10/16	62.50			
Workers wear specific boots/clothes (every week)					
Yes	6/9	66.67	0.57	0.10-3.18	0.52
No	8/15	53.33			

**Table-5:** The possible risk factors of *Salmonella* infection in the laying hens from the smart Khon Kaen University layer farm.

Factor	*Salmonella* positive/tested	Prevalence (%)	Adjusted odds ratio	95% CI	p-value
Pen floor washed (at least 2 times/month)					
Yes	3/12	25.00	0.60	0.08-4.45	0.62
No	2/12	16.67			
Layer carcasses disposal (within 24 h)					
Yes	1/8	12.50	2.33	0.22-25.25	0.49
No	4/16	25.00			
Presence of rodents at the farm (at least 3 times/month)					
Yes	2/11	18.18	0.74	0.10-5.49	0.77
No	5/13	38.46			
Changing of foot-dip (at least 3 times/month)					
Yes	1/10	10.00	3.60	0.34-38.38	0.29
No	4/14	28.57			
Having boot-disinfection bath (at least for 5 months)					
Yes	3/15	20.00	1.14	0.15-8.59	0.90
No	2/9	22.22			
Workers wear specific boots/clothes (every week)					
Yes	2/9	22.22	0.88	0.12-6.58	0.90
No	3/15	20.00			

**Table-6:** The possible risk factors of *Salmonella* contamination on the eggshells from the smart Khon Kaen University layer farm.

Factor	*Salmonella* positive/tested	Prevalence (%)	Adjusted odds ratio	95% CI	p-value
Visitors can freely enter the eggs collection area (at least 3 visitors/time/month)					
Yes	4/8	50.00	7.0	0.92-53.23	0.06
No	2/16	12.50			
Workers wear gloves before sorting eggs					
Yes	2/19	10.53	0.4	0.05-3.27	0.39
No	4/5	80.00			
Hands washed before sorting eggs					
Yes	1/14	7.14	13.0	1.20-140.73	0.04
No	5/10	50.00			
Egg sizing machine cleaned (at least 3 times/month)					
Yes	1/14	7.14	13.0	1.20-140.73	0.04
No	5/10	50.00			
Presence of flies or insects at the collection egg area (more than 3 times/month)					
Yes	5/8	62.50	25.00	2.10-298.29	0.01
No	1/16	6.25			

### Antibiotic residues in egg samples

Table-7 shows the testing detection limits of the five antibiotic groups. Table-8 shows the results of antibiotic detection using the Modified Four Plate Test for 144 egg samples. All egg albumen samples were positive for tetracyclines, sulfonamides, and aminoglycosides, with diameter inhibition zones (DIZs) in three replications of 10.38±1.16, 12.00±1.70, and 11.68±1.20 mm, respectively. In contrast, the samples were negative for penicillins, macrolides, and quinolones. The egg yolk samples were positive for aminoglycosides, quinolones, and tetracyclines at 36.11%, 11.81%, and 7.64%, respectively, with DIZs of 9.62±1.24, 8.29±0.59, and 9.67±0.71, respectively. In contrast, all the egg yolk samples were negative for sulfonamides, penicillins, and macrolides. All 144 composite eggs (e.g., albumen and yolk) were negative for penicillins and macrolides.

**Table-7:** Detection limits for different antibiotics.

Antibiotics	Concentration (μg/disc)	Inhibition zone (mm)^1^
Penicillins group	0.0320	25.67±1.15
(Penicillin G)	0.0160	22.00±0.50
	0.0080	18.67±1.61
	0.0040	15.83±1.61
	0.0020	12.00±1.00
	0.0010*	8.50±0.87
	0.0005	0.00±0.00
Tetracyclines group	0.0320	12.00±0.00
(Tetracycline)	0.0160*	9.67±0.58
	0.0080	7.67±0.58
	0.0040	0.00±0.00
	0.0020	0.00±0.00
	0.0001	0.00±0.00
Sulfonamides group	0.1000	23.67±0.58
(Sulfamethoxazole)	0.0500	20.67±0.58
	0.0250	16.00±0.00
	0.0125*	11.33±1.15
	0.0063	0.00±0.00
	0.0031	0.00±0.00
Aminoglycosides	1.0240	14.33±0.58
group (Streptomycin)	0.5120	10.67±0.58
	0.2560*	8.00±0.00
	0.1280	0.00±0.00
	0.0640	0.00±0.00
	0.0320	0.00±0.00
Macrolides group	0.0640	22.67±0.58
(Erythromycin)	0.0320	19.33±0.58
	0.0160	16.00±1.00
	0.0080	12.00±1.00
	0.0040*	9.33±1.15
	0.0020	0.00±0.00
Quinolones group	0.5120	22.67±0.58
(Enrofloxacin)	0.2560	18.67±0.58
	0.1280	15.67±0.58
	0.0640	11.33±1.15
	0.0320*	8.00±0.00
	0.0160	0.00±0.00

* Limit of detection.

1 Value±SD with triplicate

**Table-8:** The percentage of the positive antibiotic residue egg samples in six antibiotic groups tested by European Four Plate Test.

Egg type	The positive antibiotic residue (%)					

	Penicillins	Tetracyclines	Sulfonamides	Aminoglycosides	Macrolides	Quinolones
Albumen	0/144 (0.00)	144/144 (100.00)	144/144 (100.00)	144/144 (100.00)	0/144 (0.00)	0/144 (0.00)
Yolk	0/144 (0.00)	11/144 (7.64)	0/144 (0.00)	52/144 (36.11)	0/144 (0.00)	17/144 (11.81)

## Discussion

Food products can cause *Salmonella* infection in humans. In the present study, the *Salmonella* prevalence in the laying hen, eggshell, and environmental samples was higher than that found in the study by Sodagari *et al*. [3]. Our study detected no *Salmonella* in the egg samples but did detect it in the ESM eggshells at a prevalence of 15.00%. The prevalence of *Salmonella* in the eggs in the present study was lower than that found in the results of Camba *et al*. [10]. The *Salmonella* prevalence in the ESM eggshells in our study was higher than that found in the report of Adesiyun *et al*. [11] (7.7%) and Camba *et al*. [10] (0.06 %). Contaminated eggshells can cause human salmonellosis by consuming raw or undercooked eggs that lack shell-cleaning or are stored at temperatures above room temperature (30^o^C) for more than 3 weeks. The *Salmonella* contamination in eggshells in this study may have been due to cross-contamination from the *Salmonella*-positive pen floor (62.50%), a finding similar to that in Li *et al*. [1], who reported *Salmonella* cross-contamination between their laying houses and egg collection areas. In addition, eggshells in the present study could have been contaminated with *Salmonella* directly from the ESM, as our study found the prevalence of *Salmonella* in the ESM samples to be 52.50%. The main risk factor for *Salmonella* contamination in eggshells found in this study, using univariate analysis, was flies or insects at the egg collection area, followed by cleaning the ESM fewer than 3 times/month and workers not washing their hands before sorting the eggs (Table-6).

The most prevalent serovar in all the sample types in this study was *S. enterica* Corvallis, followed by Mbandaka, Braenderup, and Typhimurium. The prevalence of *Salmonella* serotypes in poultry farming depends on the sample type, study area, housing system, and associated risk factors. Corvallis exhibited the highest prevalence (60%) on the pen floors in the present study. In contrast, Mbandaka was the most prevalent serovar on the eggshells, EB, and ESM, with prevalence rates of 66.67%, 45.45%, and 47.62%, respectively. Typhimurium was the predominant serovar found in the tap water and the laying hens at 100% (2/2) and 40% (2/5), respectively. This result is consistent with the study by Pande *et al*. [12], which reported that Typhimurium isolated from laying hens commonly exhibited a higher prevalence than in the environmental samples. Recently, Typhimurium has also been reported as the main serovar found in layer farm environments (e.g., ledges, net boxes, and ventilators) [3]. Our study found two isolates of Typhimurium in the tap water outside the layer farmhouse, which may be due to *Salmonella* contamination from animal feces. S. *enterica* Corvallis is most commonly found in poultry carcasses following the slaughtering process in several countries, including Thailand [13], Brazil [14], Malaysia [15], and China [16]. In this study, the prevalence of Corvallis in laying farm environmental samples was high when compared with the results of Moraes *et al*. [17] and Camba *et al*. [18]. This may have been due to the presence of rodents at the farm (OR=25, p=0.01), as rodents can be the main reservoir of this serovar, as shown in the study by Camba *et al*. [18]. Our findings are consistent with the *S. enterica* Mbandaka prevalence found in the previous studies [19,20]. It is reportedly one of the most common serovars found in environmental samples of the dust in laying farms, including the pen floor, EB, walls, fans, and cage bottoms [19,20], with prevalence rates ranging from 27.1% to 41.7%.

The highest prevalence of antimicrobial resistance among the *Salmonella*-positive samples in this study was to tetracyclines. Only one Typhimurium isolate resisted three antibiotics (cefotaxime, enrofloxacin, and oxytetracycline). However, it can be classified as a multidrug-resistant isolate. Other *Salmonella* serovars in this study resisted only tetracycline antibiotics, which is inconsistent with the study by Zhang *et al*. [21], who reported a high multidrug resistance found in two *Salmonella* serovars of Corvallis and Mbandaka. This disagreement may be due to the isolated *Salmonella* from different sources (poultry meat). To the best of our knowledge, this is the first report of prevalent antimicrobial resistance of *Salmonella* in laying hen and environmental samples in Thailand. This study’s high *Salmonella* resistance rate to tetracycline may have been due to the frequent use or misuse of this drug in food-producing animals in Thailand [22,23]. In similar studies in Turkey [24,25] and Iran [26], *Salmonella* isolates were highly resistant to tetracycline in laying hens.

This study showed 100% tetracycline, sulfonamide, and aminoglycoside residues in egg albumen samples. The prevalence of these antibiotic residues was much higher than in egg yolk samples. In this case, the endogenous inhibitory agents from the egg albumen may have interacted with the microbial plate assay and led to false-positive samples [27]. This study found only aminoglycoside and tetracycline residues in the eggs (in both albumen and yolk). The prevalence of tetracycline residues found in this study was higher than that in the study performed by Adesiyun *et al*. [11]. In contrast, our finding was lower than that in the report by Adesiyun *et al*. [28]. This study’s most prevalent antibiotic residues were aminoglycosides, followed by tetracyclines. These findings are consistent with previous studies reporting the three highest antibiotic residues commonly found in eggs were aminoglycosides, tetracyclines, and quinolones [27,29]. These residues may be due to the contamination of feeds with these antibiotics at the feed mills, especially in commercial feeds and medicated on-farm feeds lacking information about the withdrawal requirement of these drugs. In addition, the highly prevalent tetracycline residue in eggs in the present study may have been related to the high tetracycline resistance of the *Salmonella* isolates.

### Limitations of the study

We interviewed the same interviewee each month during the 8 months of the study, which included only one layer farm. Therefore, our study may have some limitations regarding the possible risk factors of *Salmonella* contamination reported about the too wide range of 95% confidence interval of the odds values on the layer pen floor, such as the presence of rodents at the farm and the frequency of changing foot dip due to too small number of the analysis interview. These may lead to errors in the data reported and may have overestimated odds values. However, this study has shown that rodents at the farm and infrequent changing of the foot dip disinfectants are important factors contributing to *Salmonella* contamination on layer pen floor in layer farming. Another limitation was that this study used a simple *Salmonella* detection technique. *Salmonella* isolates were not molecularly identified due to resource constraints. However, we used the standard culture-based method, repeated the check using the 3M Petrifilm™ *Salmonella* Express System, and further tested serotypes for confirmation. Therefore, the identification methods may be considered acceptable.

## Conclusion

The present study showed the high prevalence of *Salmonella* contamination on the pen floors and the ESM at 62.50% (50/80) and 52.50% (21/40), respectively. In addition, the antibiotic residues of tetracycline and quinolone groups in the egg samples indicated the possible use of medicated feed and application of an inappropriate drug withdrawal time. Surveillance for *Salmonella* contamination in closed-housing systems at laying hen farms, along with good farm hygiene, and strict rodent and insect control, may reduce contamination through the dust on these farms. Further investigation is required to assess the risk factors associated with antibiotic residues in eggs, such as the requirements for workers’ knowledge about the withdrawal times of antibiotic use. Researchers should analyze antibiotic residues from feed samples and compare other conventional layer farm raising systems.

## Authors’ Contributions

PS and JA: Conceptualized and designed the study. NS and CS: Contributed to sample collection and data analysis. PS and JA: Drafted the manuscript PS: Revised the manuscript. All authors have reviewed the manuscript and approved the final version.
